# A new enzyme-linked immunosorbent assay (ELISA) for human free and bound kallikrein 9

**DOI:** 10.1186/s12014-017-9140-6

**Published:** 2017-01-17

**Authors:** Panagiota Filippou, Dimitrios Korbakis, Sofia Farkona, Antoninus Soosaipillai, Theano Karakosta, Eleftherios P. Diamandis

**Affiliations:** 1 Department of Laboratory Medicine and Pathobiology, University of Toronto, Toronto, Canada; 2 Department of Clinical Biochemistry, University Health Network, Toronto, Canada; 3 Lunenfeld-Tanenbaum Research Institute, Mount Sinai Hospital, Toronto, Canada; 4 Department of Pathology and Laboratory Medicine, Mount Sinai Hospital, Toronto, Canada; 5 Mount Sinai Hospital, Joseph & Wolf Lebovic Ctr., 60 Murray St [Box 32], Flr 6 - Rm L6-201, Toronto, ON M5T 3L9 Canada

**Keywords:** Kallikreins, Kallikrein 9, ELISA, Immunocapture, PRM, Serine protease inhibitors, a1-Antichymotrypsin, Hybrid assays

## Abstract

**Background:**

Kallikrein 9 (KLK9) is a member of the human kallikrein-related peptidases family, whose physiological role and implications in disease processes remain unclear. The active form of the enzyme is predicted to have chymotryptic activity. In the present study, we produced for the first time the active recombinant protein and monoclonal antibodies, and developed novel immunoassays for the quantification of free and bound KLK9 in biological samples.

**Methods:**

The coding sequence of mature KLK9 isoform (mat-KLK9) was expressed in an Expi293F mammalian system and the synthesized polypeptide was purified through a two-step protocol. The purified protein was used as an immunogen for production of monoclonal antibodies in mice. Hybridomas were further expanded and antibodies were purified. Newly-produced monoclonal antibodies were screened for reaction with the KLK9 recombinant protein by a state-of-the-art immunocapture/parallel reaction monitoring mass spectrometry-based methodology.

**Results:**

Anti-KLK9 antibodies were combined in pairs, resulting in the development of a highly sensitive (limit of detection: 15 pg/mL) and specific (no cross-reactivity with other KLKs) sandwich-type ELISA. Highest KLK9 protein levels were found in tonsil and sweat and lower levels in the heart, kidney and liver. Hybrid immunoassays using an anti-KLK9 antibody for antigen capture and various anti-serine protease inhibitor polyclonal antibodies, revealed the presence of an a1-antichymotrypsin-bound KLK9 isoform in biological samples.

**Conclusions:**

The ELISAs for free and bound forms of KLK9 may be highly useful for the detection of KLK9 in a broad range of biological samples, thus enabling the clarification of KLK9 function and use as a potential disease biomarker.

**Electronic supplementary material:**

The online version of this article (doi:10.1186/s12014-017-9140-6) contains supplementary material, which is available to authorized users.

## Background

Human tissue kallikrein-related peptidases (KLKs) constitute the largest family of secreted serine proteases, with diverse expression patterns and physiological roles [1]. Aberrant KLK activity has been linked to various pathologies such as respiratory diseases, neurodegeneration, skin-barrier dysfunction and cancer. Thus, KLKs are attractive targets for novel therapeutics [2]. The deregulation of KLKs at the gene and protein level has been associated with the hallmarks of cancer [3].

Human tissue kallikrein 9 (KLK9), which was originally identified as the *KLK*-*L3* gene, spans an area of 7.1 kb on chromosome 19, flanked by the *KLK8* and *KLK10* genes [4]. The full gene sequence (GenBank accession no. AF135026) contains five coding exons and the encoded KLK9 protein (UniProt accession: Q9UKQ9 (KLK9_HUMAN)) is predicted to be synthesized as a pre-pro-enzyme (1–250 amino acids) which is processed to the mat-KLK9 (lacking the signal peptide and the pro-segment) [4, 5].

According to previous RNA data, KLK9 was found to be expressed in a restricted number of tissues, including the salivary gland, ovary [4] esophagus, tonsil and skin (http://www.proteinatlas.org/). Some recent data suggest that KLK9 may play an important biological role. In brief, the mRNA level of KLK9 expression has favorable prognostic value in ovarian [6] and breast cancer [7], while elevated *KLK9* expression levels were associated with higher grade gliomas [8]. Further analysis of cancer cell lines revealed that KLK9 is constitutively expressed in breast, ovarian and lung cancer [9]. Recent studies associate *KLK9* expression patterns with non-malignant diseases, such as cardiac hypertrophy and hypertension-induced target organ damage [10] psoriatic lesions [11] and complications in asthma patients [12].

Based on the cited literature, we hypothesized that KLK9 may be involved in various pathologies and could be a disease biomarker of diagnosis/prognosis. These studies could benefit from a highly sensitive and specific KLK9 ELISA, which was not available until today.

In this study, we describe the production and characterization of mouse monoclonal antibodies against the mature KLK9 form (mat-KLK9) and the development of a highly sensitive and specific ELISA assay for the free monomer. We also developed an ELISA that measures the inhibitor-bound KLK9 form, through a hybrid assay that includes a1-antichymotrypsin antibodies. These assays were used to quantify free and bound forms of KLK9 in tissue extracts and biological fluids.

## Methods

### Production of recombinant KLK9 in the Expi293 transient mammalian expression system

The mature form of KLK9 (mat-KLK9) (aa 23–250) was expressed in the Expi293 mammalian protein expression system (ThermoFisher Scientific, Carlsbad, CA, USA). The expression plasmid pCDNA3.4, carrying the appropriate part of the KLK9 gene (pCDNA3.4-KLK9), in-frame with a mammalian IgK-chain secretion signal peptide (*METDTLLLWVLLLWVPGSTG*) was synthesized using Gene Art synthesis under optimal conditions (Invitrogen, Carlsbad, CA, USA). The pCDNA3.4-KLK9 plasmid was amplified via transformation of *E. coli* One Shot™ TOP10 chemically competent cells according to the company’s instructions (Invitrogen). The plasmid was purified (PureLink™ HiPure Plasmid Midiprep Kit, Invitrogen) and the KLK9 sequence was further confirmed by DNA sequencing (ACGT Corp. Toronto, Canada).

The mat-KLK9 protein was expressed in suspension Expi293 cells according to the manufacturer’s instructions after optimization. Briefly, for each 30 mL small scale KLK9 expression, Expi293F™ cells were diluted in Expi293™ Expression medium to a final cell density of 3 × 10^6^ cells/mL in 25.5 mL (125-mL flask). For the transfection of the Expi293F™ cells with pCDNA3.4-KLK9 plasmid: (1) 30 μg of the plasmid were diluted in Opti-MEM^®^ I Reduced Serum Medium to a total volume of 1.5 mL, (2) 90 μL of ExpiFectamine™ 293 Reagent was diluted in Opti-MEM^®^ I medium to a total volume of 1.5 mL and incubated for 5 min at room temperature, (3) The diluted DNA was added to the diluted ExpiFectamine™ 293 Reagent and the mixture was incubated for 20 min at room temperature, to allow the DNA-ExpiFectamine™ 293 Reagent complexes to form, (4) The 3 mL of the DNA-lipid complexes were added to each flask and the cells incubated at 37 °C in 8% CO_2_ air under 125 rpm shaking, (5) after 24 h incubation, a mixture of enhancers (150 μL of ExpiFectamine™ 293 Transfection Enhancer 1 and 1.5 mL of ExpiFectamine™ 293 Transfection Enhancer 2) were added to each flask (final volume: 30 mL). Media from each flask, containing the secreted KLK9 protein, were harvested at different time points (24, 48, 72 and 96 h post-transfection) and the KLK9 protein expression was verified by Western blot analysis using existing in-house KLK9 antibodies. Large scale protein expression was followed, and the media was harvested at 96 h post-transfection, concentrated 10× and stored at −80 °C until use. The yield was estimated by mat-KLK9 quantification using selected reaction monitoring (SRM) analysis (see below).

### Purification of mat-KLK9

Mat-KLK9 was purified using a two-step purification protocol. Initially the KLK9 supernatant was diluted 3 times with the equilibration buffer (50 mM Tris–HCl, pH 9.0) and the protein was purified using the Akta FPLC system on a Mono Q™ 4.6/100 PE column (GE Healthcare, Life Sciences, Mississauga, ON, Canada). The conditions of the purification procedure were as follows: Equilibration buffer: 50 mM Tris–HCl (pH 9.0), elution buffer: 50 mM Tris–HCl (pH 9.0) + 1 M NaCl, flow rate: 2 mL/min, step-gradient of the elution buffer: 5% for 10 min, linear gradient: 5–40% for 44 min (fractions of 4 mL each were collected), step-gradient: 100% for 5 min. KLK9 was eluted using linear gradient of elution buffer at approximately 200 mM NaCl. To further purify KLK9 to homogeneity, reversed-phase HPLC was used as a second purification step. KLK9-containing fractions were combined, diluted to 1% (v/v) TFA and loaded onto a Viva C4 column (5 μm, 50 × 4.6 mm, Restek, USA). The following multi-step gradient elution (0.1% trifluoroacetic acid and 0.1% trifluoroacetic acid in 90% acetonitrile) at a flow rate of 1 mL/min was used: linear gradient (10–40% acetonitrile for 5 min), 40% acetonitrile for 10 min, linear gradient (40–50% for 20 min), linear gradient (50–80% acetonitrile for 5 min), 80% for 5 min and linear gradient (80–10% acetonitrile for 2 min). KLK9 was eluted at approximately 40–50% acetonitrile. The presence and the identity of the purified KLK9 in the positive fractions (after acetonitrile evaporation) was confirmed by Western blotting and SRM analysis (see below). The protein purity was verified by silver and Coomassie-staining.

### SDS-PAGE and Western blotting analyses of KLK9

SDS-PAGE was performed using 4–12% gradient polyacrylamide gels at 200 V for 45 min (BIO-RAD). Gels were either stained using the silver staining kit (PlusOne Silver staining protein kit, GE Healthcare) or with Biosafe Coomassie staining (Invitrogen). For the Western blot analysis, a Trans-Blot Turbo Transfer Starter system (BIO-RAD) was used. Briefly, after transfer, the PVDF membranes were blocked in 5% milk for 2 h and further incubated with the in-house primary anti-KLK9 mouse monoclonal antibody in 1% milk (1/500 dilution) for 2 h. Next, membranes were washed 4 times with Tris-buffered saline, 0.1% Tween-20 (TBST) and further incubated with peroxidase-conjugated goat anti-mouse IgG antibody (1/10,000) for 45 min at room temperature. After 4 times washing with TBST, membranes were incubated with ECL Western blotting detection reagents (GE Healthcare) and exposed to X-ray film.

### Selected reaction monitoring (SRM) analysis of KLK9

Ten µg of total protein were aliquoted from a crude KLK9-secreting Expi293 cell culture supernatant, as well as from purified fractions. Samples were initially mixed with 50 mM ammonium bicarbonate (ABC) and 10 mM dithiothreitol (DTT). Following the addition of a heavy-labeled KLK9 peptide (2500 fmol for the crude supernatant and 600 fmol for the purified KLK9, respectively), samples were incubated at 60 °C for 30 min. Subsequently, 20 mM iodoacetamide (IAA) were added and samples were left in the dark for 1 h at RT. Proteins were then digested overnight at 37 °C using trypsin from porcine pancreas (Sigma, T6567-5X, USA) at 1:30 ratio (trypsin: total protein). Trypsin inactivation was accomplished with the addition of TFA at a final concentration 1% (v/v). Peptides were extracted using C18 Bond Elut OMIX tips (Agilent Technologies, Mississauga, ON, Canada) and eluted in 5 µL of 65% acetonitrile in 0.1% formic acid. Finally, eluates were further diluted to 60 μL with 0.1% formic acid. Using a 96-well microplate autosampler on an Eksigent ekspert NanoLC 425 system, 18 μL of each sample were loaded onto a 15 cm long 3 μm particle C18 analytical column (i.d. 75 μm) with an 8 μm tip (New Objective, Woburn, MA, USA). The mobile phase consisted of 0.1% formic acid in water (buffer A) and 0.1% formic acid in acetonitrile (buffer B). Peptides were separated with a 22 min gradient elution at a flow rate of 350 nL/min. The gradient started with 1% buffer B and ramped to 14% buffer B over 1 min, followed by an increase to 40% buffer B over the next 11 min. The gradient then ramped further to 65% buffer B over 2 min before it reached 100% within 1 min and was kept at that concentration for 7 min. The nano-pump was coupled online to a 6500 QTRAP 6500 quadrupole-ion trap mass spectrometer (AB Sciex, Concord, ON, Canada) equipped with a NanoSpray III source and a PhotoSpray ionization source. Declustering and entrance potentials were set to 150 and 10 V, respectively. Resolutions for both the first quadrupole and the ion trap were set to “unit” [0.7 Da Full Width at half maximum (FWHM)]. In order to exclude possible interferences, to ensure correct identity of each peak and to achieve absolute quantification of the protein, a heavy isotope-labeled peptide internal standard with a quantifying tag was synthesized for KLK9 (SpikeTides™_TQL, JPT Peptide Technologies GmbH, Berlin, Germany). For the SRM method, 4 transitions of (+3) precursor ion *VTDFFPHPGFNK* and its heavy-labelled counterpart were monitored (Additional file 1: Table S1). For each transition, scan time was set to 30 ms, while retention time was 12.1 min. Absolute quantification of the recombinant protein was achieved by comparing the peak area of the chromatographic peak of the endogenous peptide (*VTDFFPHPGFNK*) to the corresponding internal standard in each sample. Results were evaluated using the Skyline software (Mac Coss Lab Software, Seattle, WA, USA).

### Monoclonal antibody production in mice

Female BALB/c mice were purchased from Jackson laboratories via the Toronto Centre for Phenogenomics (TCP). All animal research was approved by the TCP Animal Care Committee. Mice were injected subcutaneously with 100 μg of mat-KLK9 protein, mixed (1:1) with Sigma Adjuvant System (Sigma-Aldrich). Two subsequent booster injections with 25 μg of antigen in adjuvant were performed at 3-week intervals. Final boost was an intraperitoneal injection of 25 μg of antigen in phosphate-buffered saline (137 mM NaCl, 2.7 mM KCl, 10 mM Na_2_HPO_4_, 1.8 mM KH_2_PO_4_). Three days later, the mouse spleen was excised aseptically and homogenized. Extracted spleen cells were fused with NSO murine myeloma cells (5:1 ratio) using polyethylene glycol (Sigma-Aldrich). Successfully fused cells were selected using HAT media (Invitrogen), supplemented with 20% fetal bovine serum (Hyclone, Thermo-Fisher Scientific, Waltman, MA, USA).

### Screening for immunogen-reacting clones by an IgG capture ELISA

White polystyrene 96-well microtiter plates were coated with 500 ng/well of sheep anti-mouse IgG, Fcγ fragment-specific antibody (Jackson ImmunoResearch, West Grove, PA, USA) in 50 mM Tris buffer (pH 7.8). Plates were washed 3 times with 0.05% Tween 20 in 20 mM Tris, 150 mM NaCl (pH 7.4). Cell culture supernatants of hybridoma cells diluted twofold in a solution containing 10 g/L BSA in 50 mM Tris (pH 7.8) were added to the plates and incubated for 2 h at RT with gentle shaking. Plates were then washed 3 times with the washing buffer. Five nanograms of biotinylated mat-KLK9 in assay diluent were added into each well (100 μL/well) and incubated for 2 h at room temperature (RT) with gentle shaking. Plates were washed 3 times and alkaline phosphatase-conjugated streptavidin was added (100 μL per well). Incubation was for 20 min at RT with gentle shaking, followed by a final wash (6 times). Diflunisal phosphate (DFP) solution was prepared in substrate buffer (0.1 M NaCl, 1 mM MgCl_2_ in 0.1 M Tris, pH 9.1), added to the plate (100 μL per well) and incubated for 10 min at RT with gentle shaking. Subsequently, the developing solution (1 M Tris, 0.4 M NaOH, 2 mM TbCl_3_ and 3 mM EDTA) was added on top and mixed for 1 min. Time-resolved fluorescence was measured with the Wallac EnVision 2103 Multilabel Reader (Perkin Elmer), as previously described [13].

### Expansion of hybridoma cell cultures and purification of anti-KLK9 monoclonal antibodies

Following the screening procedure, hybridoma cells were further grown and transferred in serum-free medium (CD-1 medium; Invitrogen), containing 8 mM l-glutamine. Supernatants were collected and purified using a Protein G column, according to the manufacturer’s protocol (GenScript, Piscataway NJ, USA). Culture supernatants were diluted two-fold with the binding buffer (20 mM NaH_2_PO_4_, 150 mM NaCl, pH 8.0) and loaded on the column. The column was then washed with the binding buffer and antibodies were eluted with 0.1 M glycine at pH 3.0.

### Immunocapture-PRM screening for purified mouse antibodies against KLK9

500 ng of each purified monoclonal antibody were diluted in 100 μL of coating buffer (50 mM Tris buffer, pH 7.8), added in each well of a 96-well polystyrene microtiter plate and incubated overnight at RT. The plate was then washed three times with phosphate-buffered saline (PBS). The mat-KLK9 antigen (10 or 50 ng) was added to each well and was further diluted up to 100 μL with 1% (w/v) BSA in PBS buffer. Wells with no KLK9 antigen added, were used as negative controls. The plate was incubated for 2 h at room temperature with gentle shaking and was finally washed three times with PBS and three times with 50 mM ABC. The captured antigens were subsequently subjected to trypin digestion. 88 μL of ABC buffer (100 mM), and 10 μL of DTT (50 mM) were added in each well and incubated at room temperature for 30 min. After reduction, the samples were alkylated in the dark at room temperature for 1 h, by adding 10 μL of IAA (100 mM). Five hundred femtomoles of KLK9 heavy isotope-labeled peptides with a quantitation tag (SpikeTides™_TQL, JPT Peptide Technologies GmbH, Berlin, Germany) were added to all samples prior to trypsin digestion. Each sample was digested overnight by the addition of 5 μL of sequencing grade modified trypsin (0.05 μg/μL) in 50 mM ABC (trypsin from porcine pancreas, Sigma). Trifluoroacetic acid (1%) was finally added in each well to stop the reaction. Microextraction and desalting of peptides was performed with C18, as previously described [14].

Tryptic peptides were loaded onto a 3 cm long 5 μm particle C18 trap precolumn (i.d. 200 μm) via an EASY-nLC pump (Proxeon Biosystems, Odense, Denmark) at 8 μL/min before switching in- line with the gradient. The mobile phase consisted of 0.1% of formic acid in water (buffer A) and 0.1% of formic acid in acetonitrile (buffer B). Peptides were separated on a 15 cm long 3 μm particle C18 analytical column (i.d. 75 μm) with an 8 μm tip (New Objective) with a 22 min gradient elution at a flow rate of 350 nL/min. The gradient started with 1% buffer B and ramped to 14% buffer B over 1 min, followed by an increase to 40% buffer B over the next 11 min. The gradient then ramped further to 65% buffer B over 2 min before it reached 100% within 1 min and was kept at that concentration for 7 min. The EASY-nLC system was coupled online to a Q Exactive Plus hybrid quadrupole-orbitrap mass spectrometer (Thermo Fisher Scientific, Berlin, Germany). The performance settings for the PRM method were the following: in-source collision induced dissociation was set to 3.0 eV, MS transitions in the orbitrap were acquired with 17,500 resolving power at 200 m/z, AGC target was set to 3 × 10^6^ with a maximum injection time of 100 ms, the isolation window was set to 1.0 m/z and the normalized collision energy was set to 25. The performance of the nanoLC analytical column and the mass spectrometer were verified at the beginning of each day, and every six runs thought the day, by the analysis of a quality control solution of 1 fmol/μL BSA. Two KLK9 proteotypic peptides (*WEGPEQLFR* and *LFCGATLISDR*) were used for protein identification and one peptide (*VTDFFPHPGFNK*) was used for protein quantification. The absolute quantification of the protein was calculated by taking the area ratios of heavy to light peptides and correlated to the concentration of the heavy peptides spiked into each sample. The .raw files of PRM runs were recorded for each sample and were analyzed using Skyline Targeted Proteomics Environment v3.1.0.7382 (MacCoss Lab Software, Seattle, WA, USA) and the.csv files with peptide areas were extracted.

### Development of a fluorometric KLK9 immunoassay

A sandwich type ELISA immunoassay was developed using mouse monoclonal antibodies as capture and biotinylated detection antibodies, respectively. White polystyrene microtiter plates were coated with 500 ng of mouse monoclonal anti-KLK9 antibody diluted in 50 mM Tris buffer (pH 7.8). The plate was incubated overnight at room temperature (RT) and the following day was washed 3 times with washing buffer (20 mM Tris, 150 mM NaCl, 0.05% Tween-20, pH 7.4). KLK9 standards diluted in 6% BSA or samples were then added into each well (50 μL/well) along with 50 μL of assay Buffer A (60 g/L BSA, 25 mL/L normal mouse serum, 100 mL/L normal goat serum, and 10 g/L bovine IgG in 50 mM Tris, pH 7.8, 0.005% (v/v) Tween-20) and incubated for 2 h with shaking. The plates were washed 3 times and 100 μL of the detection mouse biotinylated anti-KLK9 antibody, diluted in Buffer A (0.2 μg/mL) was applied in each well and incubated for 1 h. After washing 3 times, 100 μL of alkaline phosphatase-conjugated streptavidin (SA-ALP) was added in the wells (diluted 1/20,000 in 6% BSA) and incubated for 15 min. After 6 times final washing, 100 μL of Diflunisal phosphate (DFP) diluted in substrate buffer, were added to each well and incubated for 10 min. 100 μL of developing solution were added, mixed for 1 min and the fluorescence was measured with the Wallac EnVision 2103 Multilabel Reader (Perkin Elmer, Waltham, MA, USA).

### Hybrid immunoassays using antibodies against KLK9 and common serine protease inhibitors

Microtiter plates were coated with 28ED436 anti-KLK9 antibody (500 ng) diluted in 50 mM Tris buffer (pH 7.8) and incubated overnight. On the following day, plates were washed 3 times and 100 µL of tissue extracts/fluids diluted in Buffer B (60 g/L BSA, 25 mL/L normal mouse serum, 100 mL/L normal goat serum, 10 g/L bovine IgG, 0.005% (v/v) Tween-20 in 50 mM Tris, pH 7.8, 0.5 M KCl) were added in each well, in a total of 4 duplicates, and incubated at RT (2 h). Subsequently, plates were washed 3 times and 100 µL of polyclonal antibodies against different serine protease inhibitors [αlpha-1-antitrypsin pAb (biot)(A1AT), antithrombin III pAb (biot)(ATBIII), a1-antichymotrypsin (serpinA3 rabbit Ab), alpha-2-antiplasmin Ab (A2AP) (Fitzgerald, Acton, MA, USA), prepared in Buffer B, were added to the plates. Additionally, an equal amount of the biotinylated anti-KLK9 antibody 4ED28.2 was added in additional identical wells as a control. After 1 h incubation, plates were washed 3 times. Due to limitations in the available detection Abs, we followed both fluorogenic and colorimetric detection procedures. In more detail, for the 3 biotinylated Abs (4ED28.2, anti-AIAT and anti-ATBIII) 100 μL of alkaline phosphatase-conjugated streptavidin (SA-ALP) prepared in 6% BSA was added in each white well and incubated for 15 min. Additionally, for the rabbit anti-SERPINA3 Ab, 100 µL of the ALP-conjugated goat anti-rabbit IgG, diluted in Buffer B, were added in clear wells and incubated for 30 min at RT. Then, plates were washed 6 times and 100 μL of Diflunisal phosphate (DFP) solution, prepared in substrate buffer described above, were added into the plate (100 μL per well) and incubated for 10 min at RT with gentle shaking. Subsequently, the developing solution described above, was added on top and mixed for 1 min. Time-resolved fluorescence was measured with the Wallac EnVision 2103 Multilabel Reader (Perkin Elmer).

In the case of A2AP, 100 μL of HRP-conjugated goat anti-mouse IgG (Fc Fraction; Jackson ImmunoResearch) (in 1% milk/PBST), were added to each clear well. Following a final wash (3 times), 100 μL of 3,3,5,5′-tetramethylbenzidine substrate solution were added and plates were incubated for 15 min at 37 °C with gentle shaking. 50 μL of stop solution (2 M H_2_SO_4_) were added on top. Absorbance was measured with the Wallac EnVision 2103 Multilabel Reader (Perkin Elmer, Waltham, MA, USA) at 450 nm, with a reference wavelength of 540 nm.

### Biological fluids and tissue extracts

Our protocols for the analysis of human tissues and fluids have been approved by the Ethics Committee of Mount Sinai Hospital, Toronto, Canada. The following human tissues (fetal and adult) were used from different individuals; post-mortem. Lung, liver, kidney, heart, fat, uterus, pituitary, bone marrow, esophagus, colon, aorta, trachea, prostate, thymus, tonsil, pancreas, salivary gland, skin, stomach, small intestine, larynx, thyroid, breast, gallbladder. Tissue cytosolic extracts were prepared from snap-frozen tissues as described elsewhere [15, 16]. The supernatants representing the tissue extracts were collected and stored at −80 °C until use. The biological fluids used were: amniotic fluid of 18 weeks gestation, sweat (collected after strenuous exercise), breast milk, ascites from ovarian cancer, and synovial fluid. Fluids were stored at −20 °C until use.

### Fractionation of biological samples by size exclusion chromatography

One normal tonsil cytosolic extract and one ascites fluid sample, obtained from an ovarian cancer patient, were loaded through a 500 µL loop on a silica-based TSKGEL G3000SW gel filtration column (60 cm × 7.5 mm ID), connected to an Agilent 1100 series HPLC system. The tonsil extract was diluted 2 times in a buffer containing 0.1 M NaH_2_PO_4_ and 0.1 M Na_2_SO_4_ (pH 6.8), while the ascites sample was diluted 10 times in the same buffer. Separation was accomplished during a 60 min run at a flow-rate of 0.5 mL/min. 1 mL fractions were collected throughout the run and were analyzed for the presence of free and ACT-bound KLK9 isoforms by ELISA, as described above.

## Results

### Mat-KLK9 protein production, purification and mass spectrometric analysis

**Fig. 1:**
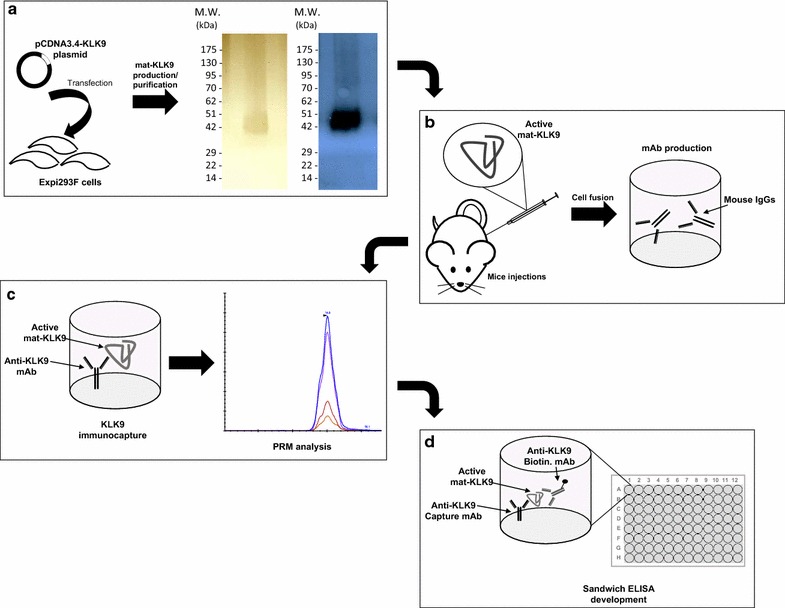
Experimental outline. **a** Mammalian Expi293F cells were transfected with a pCDNA3.4-KLK9 plasmid and used to produce mat-KLK9. Recombinant mat-KLK9 was purified with a 2-step chromatographic separation (anion-exchange followed by reverse-phase chromatography). KLK9 purity was assessed by silver staining (*left panel*) and western blot; the major KLK9 band is around 42 kDa. **b** Mice were immunized for production of anti-KLK9 monoclonal antibodies. **c** An immunocapture-PRM assay was utilized for the optimal selection of high affinity anti-KLK9 mAbs. **d** Purified monoclonal antibodies were used in pairs for the development of a sandwich type ELISA. PRM, Parallel reaction monitoring

Mat-KLK9 expression after enhancer induction was verified by Western blot analysis using an anti-KLK9 mouse monoclonal antibody that was previously produced in-house. SRM analysis using a heavy-labeled peptide for protein quantification (Additional file 1: Table S1) showed that the Expi293-KLK9 cells reached a protein yield of ~10–11 mg/L, 96 h post-transfection. As depicted in Fig. 1, the expressed protein migrated around ~42 kDa, corresponding to the glycosylated KLK9 form. Upon deglycosylation by the PNGase F, the 42 kDa band migrated at ~25 kDa (data not shown). Large scale KLK9 expression and collection of the supernatant 96 h post-transfection, was then performed, to purify the produced mat-KLK9 (aa 23–250). The recombinant mat-KLK9 was purified using 2 steps: anion-exchange chromatography, followed by reversed-phase chromatography. The purity was assessed by Coomassie and silver staining SDS-PAGE (Fig. 1a). The identification and quantification of KLK9 in the different chromatographic fractions was confirmed by SRM analysis.

### Production of mouse mAbs and screening against mat-KLK9 using immunocapture-PRM

Purified mat-KLK9 was injected into mice for the production of monoclonal antibodies by somatic cell fusion of murine splenocytes with murine myeloma cells. Eighteen hybridomas were positive after the initial round of screening and were further expanded in serum-free media. Purified IgGs were tested against mat-KLK9 protein by an immunocapture-PRM assay, according to which the 28ED436 mAb exhibited the highest binding signal for the antigen (Additional file 2: Figure S1A). We also tested 16 additional mouse monoclonal antibodies, generated by a previous in-house immunization and fusion by using a mammalian pro-KLK9 form [17] (Additional file 2: Figure S1B). Two mAbs (4ED28.2 and MIGI EII), showed high reactivity against mat-KLK9, but still, the newly produced 28ED436 antibody showed the highest KLK9 binding signal (Additional file 2: Figure S1B). Repeatability of the immuno-PRM method and system stability were assessed for all monoclonal antibodies tested. Each sample was analyzed in duplicates and the variation of light/heavy peptide ratio was estimated <13% for antibodies of higher binding affinity (e.g. 4ED28.2: CV = 12%; 28ED436: CV = 6%). Additionally, the variation of the spiked heavy peptides’ total ion current across all samples was estimated <20%.

### Development of KLK9 ELISA

The three aforementioned mAbs were tested in a mono–mono ELISA assay format against two different recombinant KLK9 forms, the mature KLK9 (mat-KLK9) (purified here) and a commercially available mammalian pro-KLK9 (R&D Systems, Minneapolis, MN, USA). According to these results (Additional file 3: Figure S2), 28ED436 was chosen as the capture Ab and 4ED28.2 as the biotinylated detection antibody.

We tested this assay against the rest of the kallikreins (KLK1–KLK15), to exclude the possibility of cross-reaction. None of the other kallikreins showed measurable readings, even at concentrations of 1 mg/L (cross-reactivity: <0.05%). Next, the limit of blank (LOB) was estimated at 10 pg/mL (matrix was 6% BSA) and the limit of detection (LOD) was around 15 pg/mL (LOD = LOB + 1.64 * SD). Linearity was assessed by diluting mat-KLK9 in BSA and the assay was linear in the range 0.023–6 ng/mL with each point displaying adequate accuracy (Additional file 4: Figure S3A). Within-run (*N* = 10) and between-run (*N* = 10) imprecision was assessed over one and 7 different days, respectively. Within-run imprecision was <12% and between-run imprecision was <21% within the measurement range (Additional file 5: Table S4). The limit of quantification (LOQ) was equal to the KLK9 concentration with ≤15% CV (20 pg/mL). The stability of native KLK9 in sweat, as well as the recombinant protein’s (mat-KLK9) stability spiked in serum (5 ng/mL), were determined in a 7-day (d) experiment (d0 → d1 → d7), during which sample aliquots were stored at 4 °C, RT and −20 °C (Additional file 4: Figure S3B). KLK9 concentration in sweat was stable after 24 h in all cases. On day seven, the KLK9 concentration decreased at RT only, by about 40% (Additional file 4: Figure S3B). KLK9 concentration spiked in serum decreased by about 60% after 24 h incubation (d1) at all conditions and remained relatively stable at 7 days (Additional file 4: Figure S3C). The KLK9 rapid decrease in serum may be attributed to the formation of heterocomplexes between the added mat- KLK9 and endogenous serum serine protease inhibitors. This is supported by additional data of spiking recombinant KLK9 proteins in serum samples obtained from female and male individuals. The mat- and pro-KLK9 forms were spiked in serum samples (at final concentrations of 5 and 10 μg/L) and incubated for 1 h at RT. Recovery for mat-KLK9 ranged between 20 and 25%, while pro-KLK9 recovery was around 90–100% (Additional files 6, 7: Tables S2,3).

To further evaluate the low recovery of KLK9 in our assay and validate the hypothesis that mat-KLK9 could form heterocomplexes with the anti-chymotrypsin serpinA3 inhibitor, we spiked different amounts of the human recombinant serpinA3 inhibitor into a given constant concentration of mat-KLK9 and measured the KLK9 free monomer. A gradual decrease in the measured KLK9 concentrations was observed along with increasing amounts of spiked serpinA3 (Additional file 9: Figure S5), clearly indicating the interference of this spiked inhibitor with the free KLK9 ELISA.

### Detection of KLK9 in tissue samples and biological fluids

**Fig. 2:**
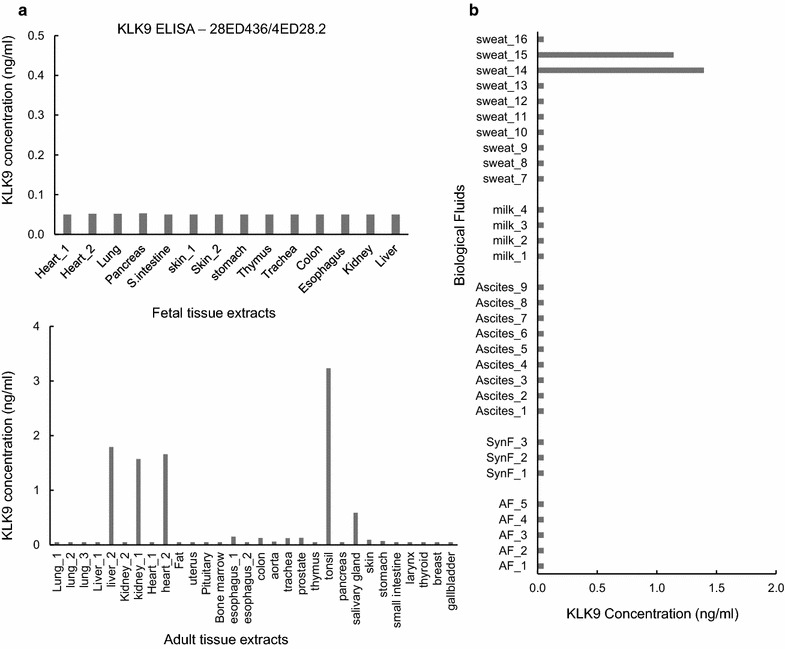
KLK9 detection in fetal and adult tissue extracts (**a**) and biological fluids (**b**) by the newly developed KLK9 ELISA. The presence of KLK9 protein was investigated in fetal (*upper panel*) and adult (*lower panel*) cytosolic tissue extracts**(a)**, as well as in biological fluids obtained from female and male subjects (**b**). KLK9 measurements are depicted in KLK9 concentration (ng/mL). KLK9 was detected in tonsil, liver, kidney and heart and in 2/10 sweat samples. For discussion see text

Using the newly developed ELISA, KLK9 protein presence was examined in various human tissue extracts (Fig. 2). In general, KLK9 ELISA signal appeared to be low in all extracts, suggesting that KLK9 might not be synthesized in abundance. Higher KLK9 levels were observed in adult tissues, compared to the fetal ones, the most positive being tonsil, kidney, liver and heart (Fig. 2a). Among the examined biological fluids, KLK9 was detected in 2/10 sweat samples, whereas the rest of the fluids demonstrated low signal (Fig. 2b).

### Detection of free and bound forms of KLK9 in biological samples

Based on our observation that mat-KLK9 had poor recovery in human serum, we sought for evidence that both free and bound forms of KLK9 exist in biological samples, similar to PSA and other proteinases. We set-up hybrid immunoassays in a mono-poly format, by using the anti-KLK9 mouse mAb 28ED436 for capture and commercially available polyclonal antibodies against several common serine protease inhibitors [such as α1-antitrypsin (A1AT), α1-antichymotrypsin (ACT), antithrombin III (ATBIII), and α2-antiplasmin (A2AP)]. We tested two KLK9-positive sweat samples (sweat samples 14 and 15; Fig. 2b). The ELISA assay signals are shown in Additional file 8: Figure S4. Elevated signal in the two sweat samples was seen with the KLK9 ELISA but not with any of the 4 hybrid assays. In ovarian cancer ascites, the major form was KLK9 bound to a1-antichymotrypsin (see also below).

### Detection of free and bound forms of KLK9 in fractionated biological samples

**Fig. 3:**
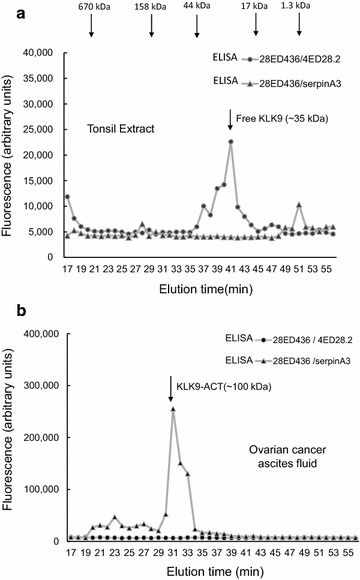
Size exclusion chromatographic separation of **a** tonsil tissue extract and **b** ovarian cancer ascites. Samples were fractionated and the collected fractions were analyzed by ELISA using the following antibody pairs: **a** mouse mAbs 28ED436 and 4ED28.2; **b** mouse mAb 28ED436 and rabbit pAb anti-serpinA3. The corresponding molecular mass of each peak was estimated according to molecular mass standards, eluting as indicated by *arrows* on *top panel*. In tonsil, the major form is free KLK9, eluting at a molecular mass of ~35 kDa. In ascites, the major form is KLK9 bound to a1-antichymotrypsin (KLK9-ACT), eluting around 100 kDa. For more discussion see text

Two samples with detectable KLK9 (tonsil extract and ascites fluid), were subjected to gel filtration chromatography and fractions analyzed with two immunoassays (KLK9 ELISA and KLK9-ACT hybrid assay). For tonsil, KLK9 ELISA had maximum signal at 41 min elution time, corresponding to a molecular mass around 40 kDa (Fig. 3a). The KLK9-ACT ELISA gave no detectable signal in any of the fractions. For ascites fluid, KLK9 ELISA gave no signal, while maximum signal with the KLK9-ACT hybrid ELISA assay was seen at 31 min (corresponding to a molecular mass of ~100 kDa) (Fig. 3b). These data suggest that some biological fluids (tissue extracts, sweat) have predominantly free KLK9 (~40 kDa) while others (ascites) contain predominantly KLK9 bound to a1-antichymotrypsin.

## Discussion

Human kallikrein 9 (KLK9) is a serine protease and a member of the kallikrein family. Its physiological and pathophysiological roles have not as yet been elucidated. Some recent data suggest that KLK9 may play an important biological role. In brief, *KLK9* gene expression was found to have favorable prognostic value in ovarian and breast cancer [6, 7], while elevated *KLK9* expression levels were associated with higher grade gliomas [8]. *KLK9* gene expression has also been related to non-malignant diseases. Recent studies associate *KLK9* expression patterns with cardiac hypertrophy and hypertension-induced target organ damage [10], psoriatic lesions [11] and complications in asthma patients [12]. In general, KLK9-related studies are still very few.

We previously produced KLK9 recombinant protein and anti-KLK9 monoclonal and polyclonal antibodies [17], which were used to develop an ELISA (mono-poly format), applied for screening a variety of biological samples [18]. However, due to the assay’s poor sensitivity, there was still a need for improved analytical tools.

Here, mature, enzymatically active mat-KLK9 was produced in mammalian cells. The purified polypeptide was used for the production of new mouse monoclonal antibodies (Fig. 1). It has been previously shown that immunoaffinity and mass spectrometric methodologies can be combined for the effective screening of hybridoma supernatants against the native forms of antigens [14] or for elucidation of antigens bound to mAbs of unknown specificity [19]. In the present study, we utilized a mass spectrometer (Q-Exactive Plus) to design PRM experiments (Additional file 2: Figure S1) and rapidly screen the monoclonal antibodies [14, 19, 20]. A potential limitation of the current protocol is perhaps the inaccuracy of antigen quantification in some cases. In more detail, the structure of the formatted immunocomplex could cause incomplete trypsin digestion, due to occlusion of cleavage sites, and thus in inadequate MS analysis. Therefore, one should consider applying more stringent sample prep conditions, such as heating above 60 °C during the initial reduction step, or even adding an extra denaturing factor (e.g. Rapigest).

A newly produced mAb (28ED436) and a pre-existing one (4ED28.2) were paired to develop a sandwich-type ELISA (Additional file 3: Figure S2). The assay was highly sensitive (LOD: 15 pg/mL), thus meeting the demands for future clinical studies [21], as well as highly specific (cross-reactivity with all other KLKs: <0.05%). The estimated recovery of mat-KLK9 from human serum was low (~20–25%), whereas pro-KLK9 recovery was much higher (90–100%), suggesting a possible interaction of the active KLK9 enzyme with endogenous inhibitors (Additional files 6, 7: Table S2, 3). This speculation was strengthened by the observation that the titration of the serpinA3 inhibitor in mat-KLK9 solution interfere with the free KLK9 ELISA (Additional file 9: Figure S5).

Screening of human tissue samples revealed higher KLK9 levels in adult tissues compared to the fetal ones, predominantly in tonsil extracts and at lower levels in kidney, liver and heart (Fig. 2a). These findings are in good agreement with RNA expression data (http://www.proteinatlas.org/). Only two out of 10 sweat samples were moderately positive for KLK9 among the tested biological fluids (Fig. 2b). KLK9 signal was very low in the sera of healthy females and males, in accordance with previous studies [16]. In light of the recovery experiments, we speculated that these findings could point to a significant binding of KLK9 by endogenous serum inhibitors [22–24]. The formation of these heterocomplexes [25] could effectively mask KLK9 epitopes. This has been observed with many other kallikreins, including PSA [24, 26, 27].

We set up hybrid assays using one capture Ab (28ED436) and polyclonal Abs targeting the most common serine protease inhibitors, such as A1AT, serpinA3, ATBIII and A_2_AP. The KLK9–serpinA3 Ab combination gave the highest signal, compared to the rest of the inhibitors. This was not surprising, given that KLK9 is predicted to be a chymotryptic enzyme [28]. Further screening of biological tissues and fluids, suggests that in some samples KLK9 is mostly present in its bound to ACT form (e.g. ascites) while in others (e.g. sweat) it is a free monomer. To strengthen our hypothesis, we sought for free and bound KLK9 forms in two biological samples (i.e. a tonsil homogenate and ascites fluid) which had been fractionated by size exclusion chromatography (Fig. 3). The results are suggestive of two distinct forms for KLK9 in each sample. In tonsil homogenate, which was only positive with the KLK9 assay, there is only one peak, appearing at ~40 kDa (Fig. 3a). In ovarian cancer ascites, that was positive only with the hybrid assay, there is only one 100 kDa form (Fig. 3b).

These data suggest that KLK9, like other kallikreins [29, 30] exists as a free active protease in tissues or fluids devoid of inhibitors (e.g. tonsil and sweat) or represents a pro-enzyme or an inactivated proteolytic form [31, 32]. Active KLK9 (mat-KLK9) also interacts with endogenous protease inhibitors in biological samples and more specifically, with a1-antichymotrypsin (ACT). In a similar manner, a1-antichymotrypsin binds to other chymotrypsin-like kallikreins, such as PSA and/or KLK7, in serum [31, 33]. Since the free/total PSA ratio in serum of prostate cancer patients is used for diagnostic and prognostic purposes [26], this could suggest a similar application of KLK9.

In summary, our newly developed tools for quantifying free and ACT-bound KLK9 with high sensitivity and specificity will facilitate further studies aiming to better define the role of KLK9 in health and disease.

## Conclusions

The development of ELISAs for the accurate quantification of the free and bound to a1-antichymotrypsin KLK9 forms will be a valuable tool for the quantification of KLK9 in a broad range of biological samples in normal and diseased individuals. Our newly developed tools for quantifying free and ACT-bound KLK9 with high sensitivity and specificity will facilitate further studies aiming to better define the role of KLK9 in health and disease.

## Additional files

**Additional file 1: Table S1.** SRM assay parameters for KLK9 analysis.

**Additional file 2: Figure S1.** Screening for KLK9 monoclonal antibodies by immunocapture-PRM. **(A)** Microtiter plates were coated with equal amounts of purified mAbs and incubated with 50 ng of mat-KLK9. Following Ag capture, the proteins in the wells were trypsin-digested and peptides were analyzed by a PRM assay. Results are depicted as KLK9 peptide intensity ratios (Light-to-Heavy, L/H) with bars representing the respective standard error. **(B)** Comparison of signals between the newly produced mAb 28ED436 and 16 previously developed mAbs against mat-KLK9. Trypsin digestion and PRM analysis were performed as in panel A. The three highest affinity antibodies were used for KLK9 ELISA development.

**Additional file 3: Figure S2.** Testing mouse anti-KLK9 mAbs for optimal pairing against KLK9. Standard sandwich ELISAs were developed using the following combinations: (i) 28ED436 *vs.* biotinylated 4ED28.2 and MIGI EII, (ii) 4ED28.2 *vs.* biotinylated 28ED436 and MIGI EII, and (iii) MIGI EII *vs.* biotinylated 28ED436 and 4ED28.2. The mat-KLK9 and the pro-form of KLK9 (R&D systems) (final concentrations: 2.5 and 5 μg/L) were used as antigens. The best signal was obtained with the pair 28ED436 (coating)-4ED28.2 (biotinylated; detection). For more details see text. 1. pro-KLK9 (5 μg/L), 2. pro-KLK9 (2.5 μg/L), 3. mat-KLK9 (5 μg/L), 4. mat-KLK9 (2.5 μg/L).

**Additional file 4: Figure S3.** Linearity and stability of the KLK9 immunoassay. **(A)** The linearity of KLK9 ELISA was assessed by diluting recombinant KLK9 in BSA. Serial dilutions of the samples were prepared and the assay was performed by following the described protocol. Linear correlation was estimated between the theoretically spiked KLK9 concentrations and the ELISA-estimated KLK9 concentrations (regression coefficient β1 = 1.20, P < 0.0001). Sample stability was tested through a 7-day experiment, by storing sweat samples **(B)**, as well as serum samples with spiked mat-KLK9 **(C)** at room temperature (RT), 4 °C and −20 °C. KLK9 was measured at 3 points (Days 0, 1 and 7) by the ELISA assay. For comments see text.

**Additional file 5: Table S4.** ELISA within-run and total imprecision.

**Additional file 6: Table S2.** Recovery of recombinant mat-KLK9 spiked in female (F) and male (M) serum samples.

**Additional file 7: Table S3.** Recovery of recombinant pro-KLK9 spiked in female (F) and male (M) serum samples.

**Additional file 8: Figure S4.** Development of hybrid ELISAs for the detection of KLK9 heterocomplexes with serine protease inhibitors. In-house generated mAb 28ED436 was used as capture Ab, while pAbs against common serine protease inhibitors (e.g. A1AT, ACT, ATBIII, and A2AP) were used as secondary Abs in two biological fluids (sweat 14 and 15). The A2AP (HRP-conjugated) levels were undetectable and are not shown in the graph. KLK9 measurements were depicted as arbitrary fluorescence units. The ELISAs used include the first antibody as coating and the second antibody for detection. Note detection of free KLK9 in 2 sweat samples (↓). For more discussion see text.

**Additional file 9: Figure S5.** KLK9 free monomer detection by the newly developed KLK9 ELISA upon the formation of serpinA3–KLK9 heterocomplexes. Mat-KLK9 (0.5 μg) was incubated either alone (control) or with different amounts of the human recombinant serpinA3 inhibitor (R&D systems) [at molar ratios (KLK9/SerpinA3): 1/0.2, 1/0.5, 1/1 and 1/2] in 50 mM Tris–HCl (pH 8.0) for 1 h at 37 °C. The samples were further diluted with 6% BSA and the assay was performed by following the described protocol (see “Methods” section). The values of the sample containing no inhibitor (control) was arbitrarily defined as 100 % recovery. The samples containing serpinA3–KLK9 complexes were expressed as % recovery of KLK9 compared to the control.

## Abbreviations

A1AT: a1-antitrypsin
ATB-III: antithrombin-III
A2AP: alpha-2 antiplasmin
ACT: a1-antichymotrypsin
mAb: monoclonal antibody
PRM: parallel reaction monitoring
KLK: kallikrein
KLK9: kallikrein 9
pAb: polyclonal antibody
RT: room temperature
ABC: ammonium bicarbonate
DTT: dithiothreitol
IAA: iodoacetamide
TFA: trifluoroacetic acid
SRM: selected reaction monitoring
SDS-PAGE: sodium dodecyl sulphate-polyacrylamide gel electrophoresis
HRP: horseradish peroxidase
AF: amniotic fluid
SA-ALP: alkaline phosphatase-conjugated streptavidin
DFP: diflunisal phosphate
SynF: synovial fluid
TBS: Tris-buffered saline
PBS: phosphate-buffered saline
SD: standard deviation
LOB: limit of blank
LOD: limit of detection
LOQ: limit of quantification
AMC: 7-amino-4-methylcoumarin
