# Supplementary material for: A new enzyme-linked immunosorbent assay (ELISA) for human free and bound kallikrein 9
Source: Clin Proteomics. 2017 Jan 17;14:4. doi: 10.1186/s12014-017-9140-6 (PMC5241945; doi:10.1186/s12014-017-9140-6)
Supplement: Supplementary file 1 — Additional file 1: Table S1. SRM assay parameters for KLK9 analysis. [file 12014_2017_9140_MOESM1_ESM.docx]

Table S1. SRM assay parameters for KLK9 analysis.

| **UniProtKB/**  **Swiss-Prot protein name** | **Uniprot Entry number** | **Peptide Sequence** | **Natural Peptide Q1 (m/z)** | **Precursor Charge** | **Natural Product Q3 (m/z)** | **Fragment Charge** | **Fragment Ion type** | **Collision Energy (V)** | **Retention Time (min)** | **Scan Time (ms)** |
| --- | --- | --- | --- | --- | --- | --- | --- | --- | --- | --- |
| **Kallikrein-9** | Q9UKQ9 | **VTDFFPHPGFNK** | 469.2348 | +++ | 796.41 | + | y7 | 23.1 | 12.1 | 30 |
|  |  |  |  |  | 562.2984 | + | y5 | 23.1 | 12.1 | 30 |
|  |  |  |  |  | 653.8144 | ++ | y11 | 23.1 | 12.1 | 30 |
|  |  |  |  |  | 610.2871 | + | b5 | 23.1 | 12.1 | 30 |
|  |  | **VTDFFPHPGFNK(Heavy**) | 471.9062 | +++ | 804.4242 | + | y7 | 23.1 | 12.1 | 30 |
|  |  |  |  |  | 570.3126 | + | y5 | 23.1 | 12.1 | 30 |
|  |  |  |  |  | 657.8215 | ++ | y11 | 23.1 | 12.1 | 30 |
|  |  |  |  |  | 610.2871 | + | b5 | 23.1 | 12.1 | 30 |
