# Supplementary figures and images for: A new enzyme-linked immunosorbent assay (ELISA) for human free and bound kallikrein 9
Source: Clin Proteomics. 2017 Jan 17;14:4. doi: 10.1186/s12014-017-9140-6 (PMC5241945; doi:10.1186/s12014-017-9140-6)

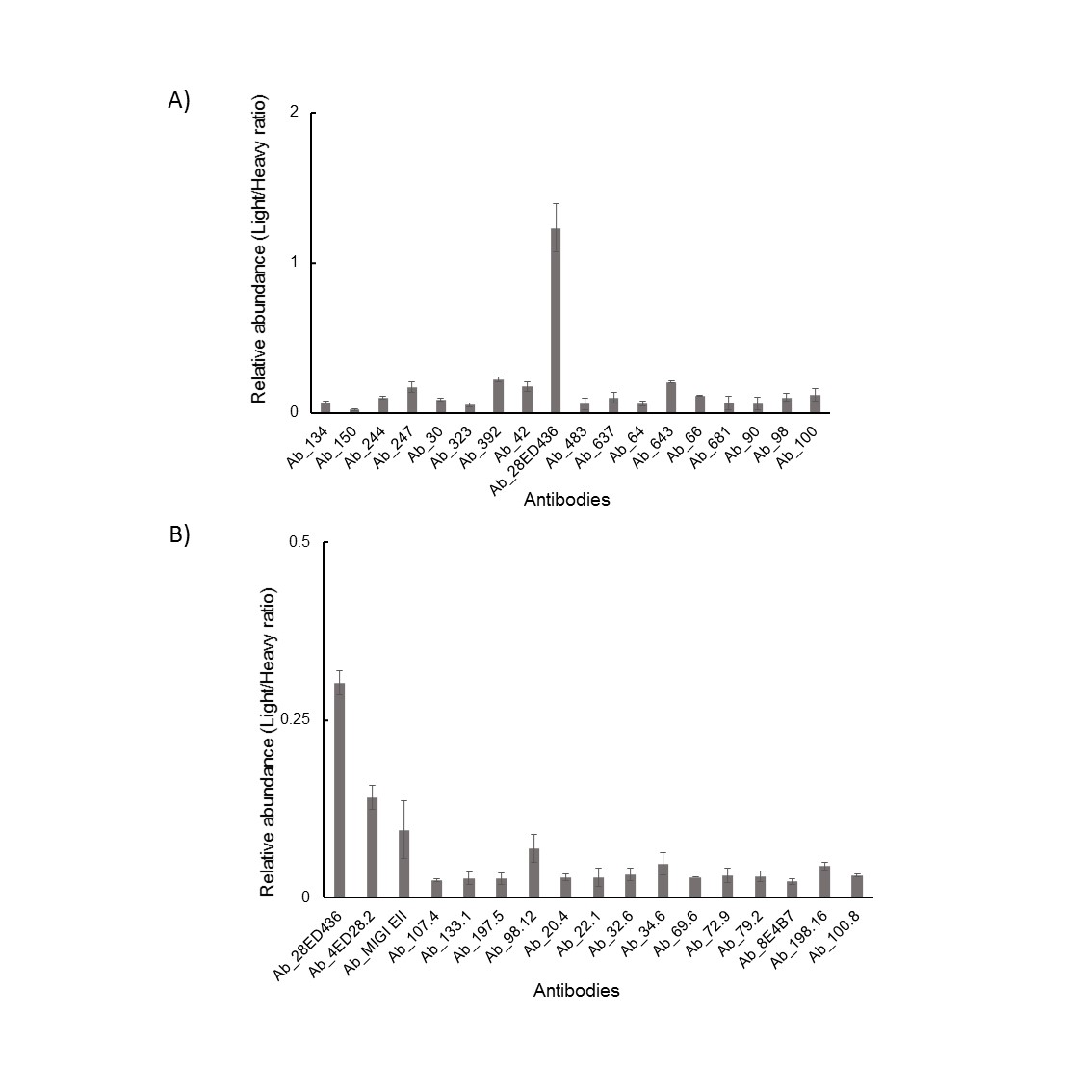

Supplement: Supplementary file 2 — Additional file 2: Figure S1. Screening for KLK9 monoclonal antibodies by immunocapture-PRM. (A) Microtiter plates were coated with equal amounts of purified mAbs and incubated with 50 ng of mat-KLK9. Following Ag capture, the proteins in the wells were trypsin-digested and peptides were analyzed by a PRM assay. Results are depicted as KLK9 peptide intensity ratios (Light-to-Heavy, L/H) with bars representing the respective standard error. (B) Comparison of signals between the newly produced mAb 28ED436 and 16 previously developed mAbs against mat-KLK9. Trypsin digestion and PRM analysis were performed as in panel A. The three highest affinity antibodies were used for KLK9 ELISA development. [file 12014_2017_9140_MOESM2_ESM.docx]

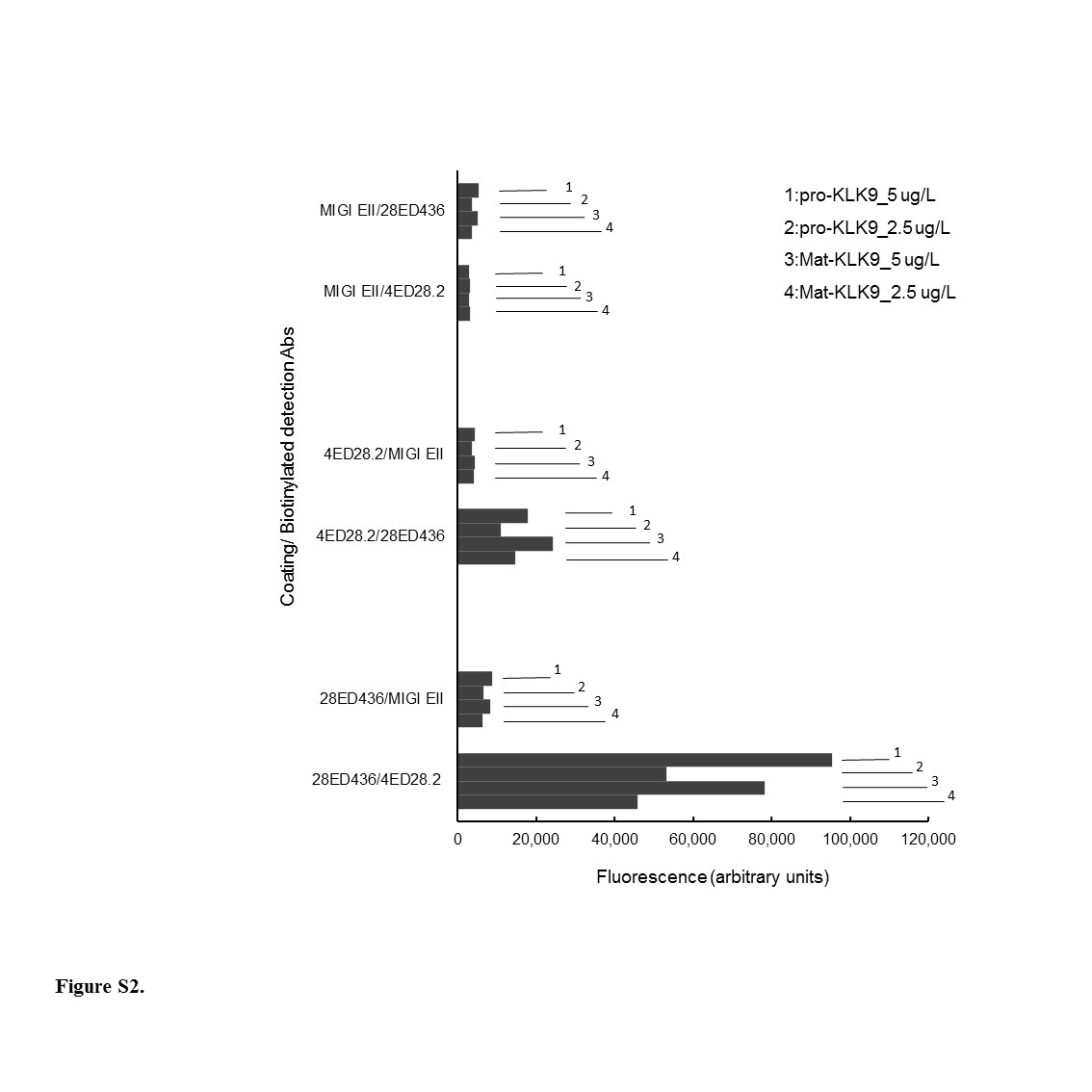

Supplement: Supplementary file 3 — Additional file 3: Figure S2. Testing mouse anti-KLK9 mAbs for optimal pairing against KLK9. Standard sandwich ELISAs were developed using the following combinations: (i) 28ED436 vs. biotinylated 4ED28.2 and MIGI EII, (ii) 4ED28.2 vs. biotinylated 28ED436 and MIGI EII, and (iii) MIGI EII vs. biotinylated 28ED436 and 4ED28.2. The mat-KLK9 and the pro-form of KLK9 (R&D systems) (final concentrations: 2.5 and 5 μg/L) were used as antigens. The best signal was obtained with the pair 28ED436 (coating)-4ED28.2 (biotinylated; detection). For more details see text. 1. pro-KLK9 (5 μg/L), 2. pro-KLK9 (2.5 μg/L), 3. mat-KLK9 (5 μg/L), 4. mat-KLK9 (2.5 μg/L). [file 12014_2017_9140_MOESM3_ESM.docx]

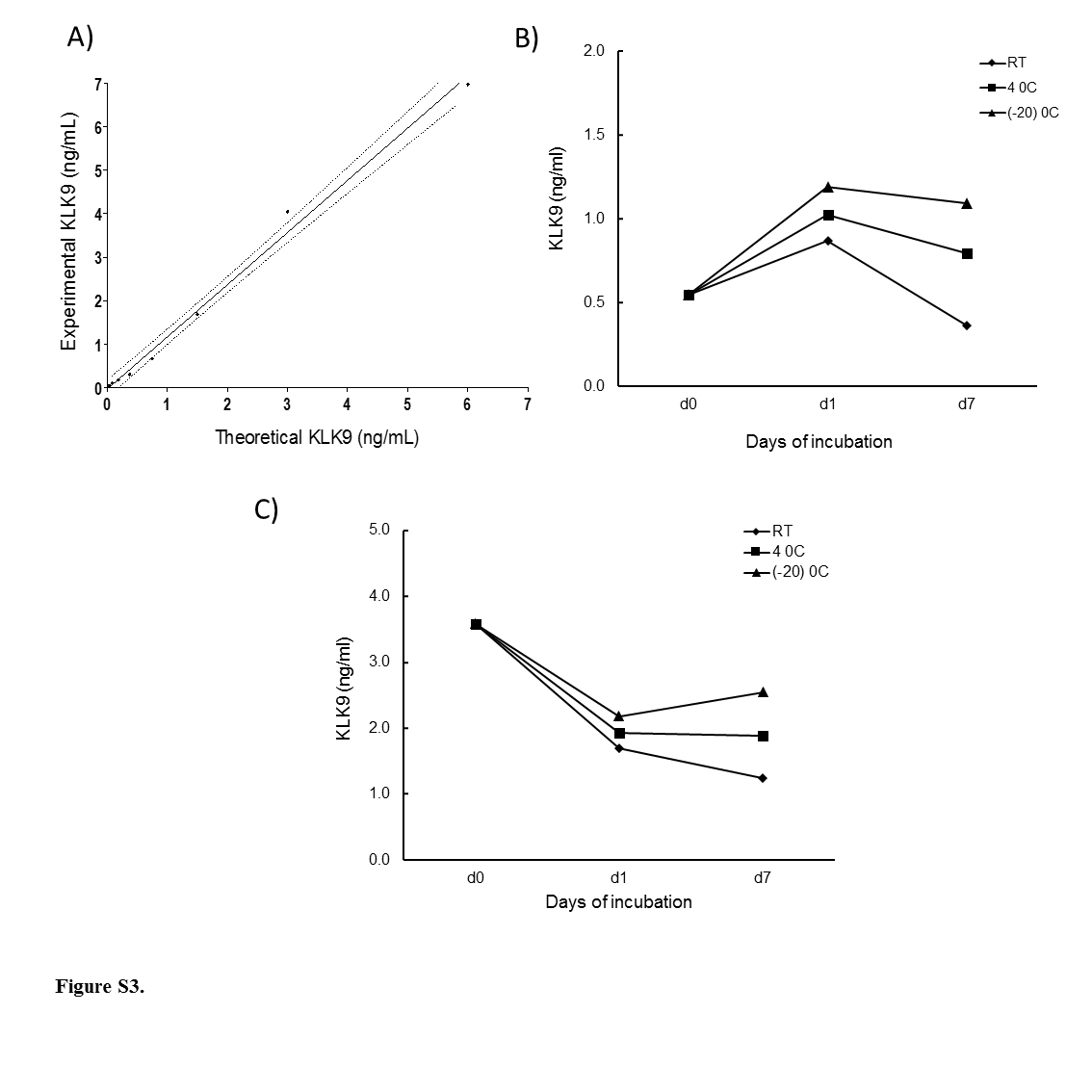

Supplement: Supplementary file 4 — Additional file 4: Figure S3. Linearity and stability of the KLK9 immunoassay. (A) The linearity of KLK9 ELISA was assessed by diluting recombinant KLK9 in BSA. Serial dilutions of the samples were prepared and the assay was performed by following the described protocol. Linear correlation was estimated between the theoretically spiked KLK9 concentrations and the ELISA-estimated KLK9 concentrations (regression coefficient β1 = 1.20, P < 0.0001). Sample stability was tested through a 7-day experiment, by storing sweat samples (B), as well as serum samples with spiked mat-KLK9 (C) at room temperature (RT), 4 °C and −20 °C. KLK9 was measured at 3 points (Days 0, 1 and 7) by the ELISA assay. For comments see text. [file 12014_2017_9140_MOESM4_ESM.docx]

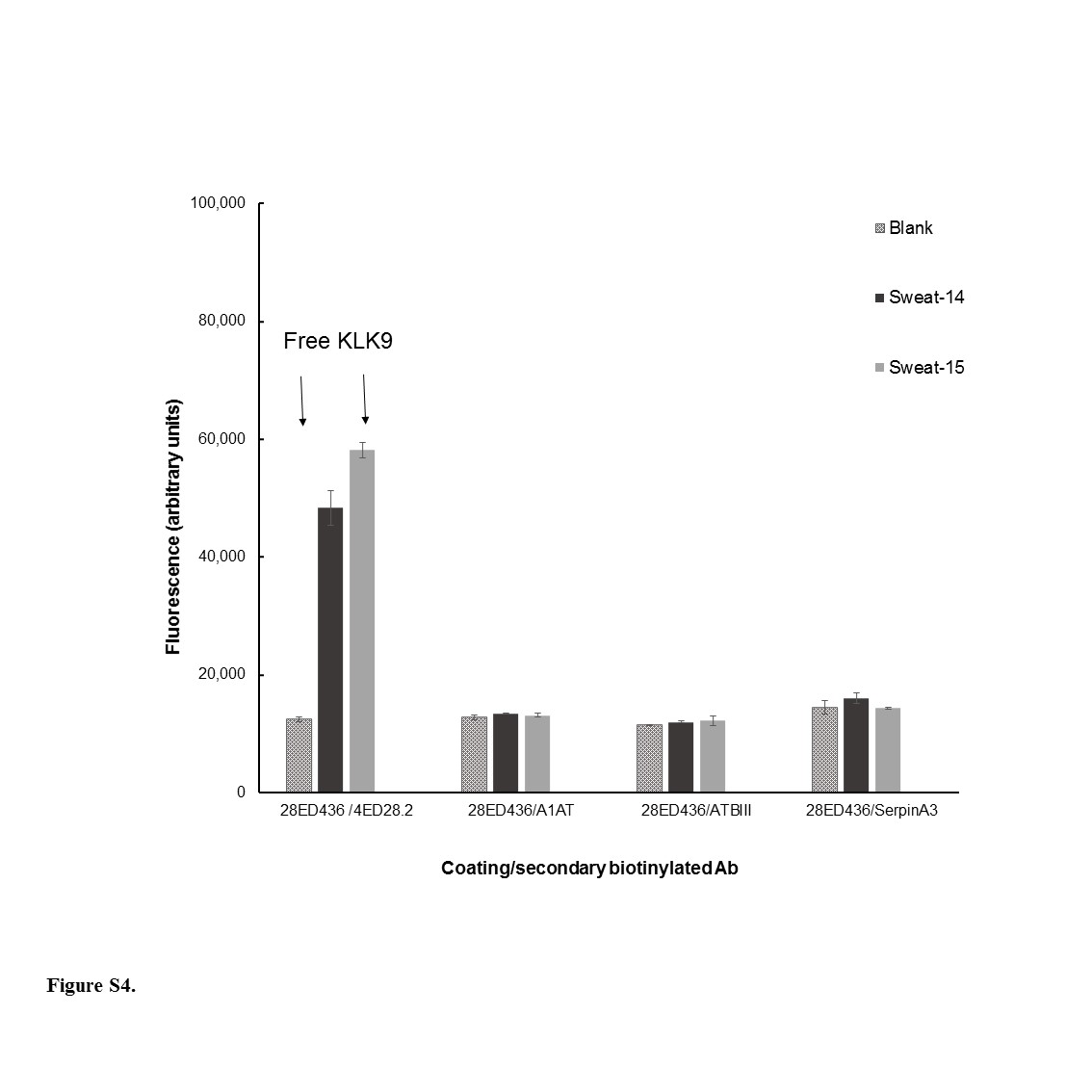

Supplement: Supplementary file 8 — Additional file 8: Figure S4. Development of hybrid ELISAs for the detection of KLK9 heterocomplexes with serine protease inhibitors. In-house generated mAb 28ED436 was used as capture Ab, while pAbs against common serine protease inhibitors (e.g. A1AT, ACT, ATBIII, and A2AP) were used as secondary Abs in two biological fluids (sweat 14 and 15). The A2AP (HRP-conjugated) levels were undetectable and are not shown in the graph. KLK9 measurements were depicted as arbitrary fluorescence units. The ELISAs used include the first antibody as coating and the second antibody for detection. Note detection of free KLK9 in 2 sweat samples (↓). For more discussion see text. [file 12014_2017_9140_MOESM8_ESM.docx]

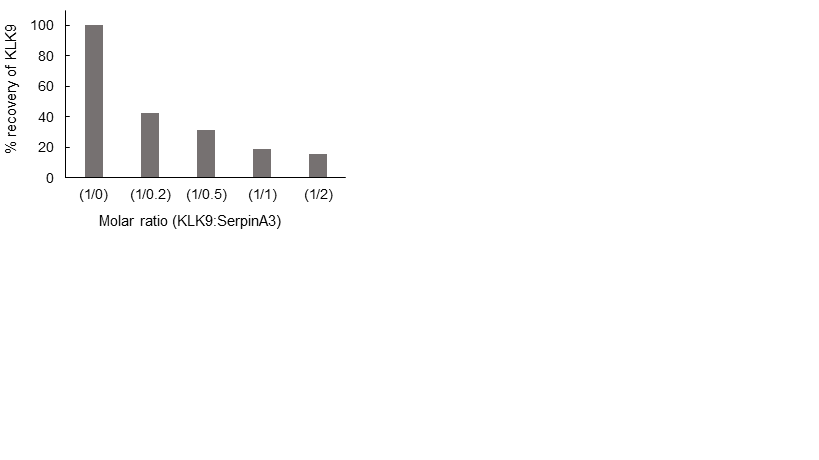

Supplement: Supplementary file 9 — Additional file 9: Figure S5. KLK9 free monomer detection by the newly developed KLK9 ELISA upon the formation of serpinA3–KLK9 heterocomplexes. Mat-KLK9 (0.5 μg) was incubated either alone (control) or with different amounts of the human recombinant serpinA3 inhibitor (R&D systems) [at molar ratios (KLK9/SerpinA3): 1/0.2, 1/0.5, 1/1 and 1/2] in 50 mM Tris–HCl (pH 8.0) for 1 h at 37 °C. The samples were further diluted with 6% BSA and the assay was performed by following the described protocol (see “Methods” section). The values of the sample containing no inhibitor (control) was arbitrarily defined as 100 % recovery. The samples containing serpinA3–KLK9 complexes were expressed as % recovery of KLK9 compared to the control. [file 12014_2017_9140_MOESM9_ESM.docx]
