# Supplementary material for: A new enzyme-linked immunosorbent assay (ELISA) for human free and bound kallikrein 9
Source: Clin Proteomics. 2017 Jan 17;14:4. doi: 10.1186/s12014-017-9140-6 (PMC5241945; doi:10.1186/s12014-017-9140-6)
Supplement: Supplementary file 5 — Additional file 5: Table S4. ELISA within-run and total imprecision. [file 12014_2017_9140_MOESM5_ESM.docx]

Table S4. ELISA within-run and total imprecision.

| **Within-run** | | |
| --- | --- | --- |
| **KLK9 levels**  **(ng/mL)^1^** | **SD^2^** | **CV (%)** |
| 10.0 | 0.59 | 6.2 |
| 5.0 | 0.17 | 3.3 |
| 2.5 | 0.027 | 1.1 |
| 1.25 | 0.046 | 3.6 |
| 0.625 | 0.018 | 2.8 |
| 0.3125 | 0.014 | 4.6 |
| 0.156 | 0.009 | 5.8 |
| 0.078 | 0.003 | 4.1 |
| 0.039 | 0.003 | 8.6 |
| 0.0195 | 0.002 | 12.1 |
| **Between-run** | | |
| **KLK9 levels**  **(ng/mL)** | **SD** | **CV (%)** |
| 10.0 | 0.52 | 5.4 |
| 5.0 | 0.2 | 3.8 |
| 2.5 | 0.03 | 1.3 |
| 1.25 | 0.04 | 3.2 |
| 0.625 | 0.014 | 2.3 |
| 0.3125 | 0.03 | 10.7 |
| 0.156 | 0.007 | 4.5 |
| 0.078 | 0.003 | 3.6 |
| 0.039 | 0.003 | 8.5 |
| 0.0195 | 0.004 | 21.0 |

1. ELISA used was for KLK9, with monoclonal antibodies 28ED436 and 4ED28.2. For more details and discussion see text.
2. Standard deviation (SD).
