# Supplementary material for: A new enzyme-linked immunosorbent assay (ELISA) for human free and bound kallikrein 9
Source: Clin Proteomics. 2017 Jan 17;14:4. doi: 10.1186/s12014-017-9140-6 (PMC5241945; doi:10.1186/s12014-017-9140-6)
Supplement: Supplementary file 6 — Additional file 6: Table S2. Recovery of recombinant mat-KLK9 spiked in female (F) and male (M) serum samples. [file 12014_2017_9140_MOESM6_ESM.docx]

Table S2. Recovery of recombinant mat-KLK9 spiked in female (F) and male (M) serum samples.

| **Serum samples** | **Spiked KLK9**  **(ng/ml)^1^** | **Measured KLK9 (ng/ml)^2^** | **% Recovery** |
| --- | --- | --- | --- |
| **F1** | 5.0 | 1.06 | 21 |
| **F2** | 5.0 | 1.09 | 22 |
| **F4** | 5.0 | 1.07 | 21 |
| **M1** | 5.0 | 0.91 | 18 |
| **M3** | 5.0 | 0.96 | 19 |
| **M5** | 5.0 | 1.12 | 22 |
| **F1** | 10.0 | 2.3 | 23 |
| **F4** | 10.0 | 2.42 | 24 |
| **M1** | 10.0 | 2.24 | 22 |
| **M3** | 10.0 | 2.17 | 22 |
| **M5** | 10.0 | 2.36 | 24 |

1. Spiked KLK9 was mat-KLK9 produced in Expi293F cells.
2. ELISA used was for KLK9, with monoclonal antibodies 28ED436 and 4ED28.2. For more details and discussion see text.
