# Supplementary material for: A new enzyme-linked immunosorbent assay (ELISA) for human free and bound kallikrein 9
Source: Clin Proteomics. 2017 Jan 17;14:4. doi: 10.1186/s12014-017-9140-6 (PMC5241945; doi:10.1186/s12014-017-9140-6)
Supplement: Supplementary file 7 — Additional file 7: Table S3. Recovery of recombinant pro-KLK9 spiked in female (F) and male (M) serum samples. [file 12014_2017_9140_MOESM7_ESM.docx]

Table S3. Recovery of recombinant pro-KLK9 spiked in female (F) and male (M) serum samples.

| **Serum samples** | **Spiked pro-hK9**  **(ng/ml)^1^** | **Measured pro-hK9 (ng/ml)^2^** | **% Recovery** |
| --- | --- | --- | --- |
| **F2** | 5.0 | 5.71 | 114 |
| **F4** | 5.0 | 4.66 | 93 |
| **M1** | 5.0 | 4.53 | 91 |
| **M5** | 5.0 | 5.98 | 120 |
| **F2** | 10.0 | 9.96 | 99.5 |
| **M1** | 10.0 | 9.47 | 95 |
| **M5** | 10.0 | 10.21 | 102 |

1. pro-KLK9 was purchased from R&D systems.
2. ELISA used was for KLK9, with monoclonal antibodies 28ED436 and 4ED28.2. For more details and discussion see text.
